# Association of SARS-CoV-2 Seropositivity With Myalgic Encephalomyelitis and/or Chronic Fatigue Syndrome Among Children and Adolescents in Germany

**DOI:** 10.1001/jamanetworkopen.2022.33454

**Published:** 2022-09-27

**Authors:** Anna-Lisa Sorg, Selina Becht, Marietta Jank, Jakob Armann, Ulrich von Both, Markus Hufnagel, Fabian Lander, Johannes G. Liese, Tim Niehues, Eva Verjans, Martin Wetzke, Silvia Stojanov, Uta Behrends, Christian Drosten, Horst Schroten, Rüdiger von Kries

**Affiliations:** 1 Institute of Social Paediatrics and Adolescent Medicine, Division of Paediatric Epidemiology, Ludwig-Maximilians-University Munich, Munich, Germany; 2 University Children’s Hospital, Eberhard Karls University, Tuebingen, Germany; 3 Paediatric Infectious Diseases, Department of Paediatrics, Medical Faculty Mannheim, Heidelberg University, Mannheim, Germany; 4 Department of Pediatrics, University Hospital and Medical Faculty Carl Gustav Carus, Technical University Dresden, Dresden, Germany; 5 Dr von Hauner Children’s Hospital, University Hospital, Ludwig-Maximilians-University Munich, Munich, Germany; 6 Department of Paediatrics and Adolescent Medicine, Medical Faculty, University Medical Centre, University of Freiburg, Freiburg, Germany; 7 Department of Paediatrics, University Hospital and Medical Faculty Carl Gustav Carus, Technical University Dresden, Dresden, Germany; 8 University Hospital of Wuerzburg, Division of Paediatric Infectious Diseases, Department of Paediatrics, Wuerzburg, Germany; 9 Department of Paediatrics, Helios Klinikum Krefeld, Krefeld, Germany; 10 Department of Paediatrics, Medical Faculty, University Hospital RWTH Aachen, Aachen, Germany; 11 Centre for Paediatrics and Adolescent Medicine, Hannover Medical School, Excellence Cluster RESIST (Resolving Infection Susceptibility), Deutsche Forschungsgemeinschaft, Hannover, Germany; 12 Department of Paediatrics, Faculty of Medicine, Technical University Munich, Munich, Germany; 13 Institute of Virology, Charité Universitätsmedizin Berlin, Berlin, Germany.

## Abstract

**Importance:**

During the COVID-19 pandemic, a reduction in quality of life and physical and mental health among children and adolescents has been reported that may be associated with SARS-CoV-2 infection and/or containment measures.

**Objective:**

To assess the association of SARS-CoV-2 seropositivity with symptoms that may be related to myalgic encephalomyelitis and/or chronic fatigue syndrome (ME/CFS) among children and adolescents.

**Design, Setting, and Participants:**

This substudy of the cross-sectional SARS-CoV-2 seroprevalence surveys in Germany (SARS-CoV-2 KIDS) was performed in 9 pediatric hospitals from May 1 to October 31, 2021. Pediatric patients were recruited during an inpatient or outpatient visit regardless of the purpose of the visit. Parental questionnaires and serum samples were collected during clinically indicated blood draws. The parental questionnaire on demographic and clinical information was extended by items according to the DePaul Symptom Questionnaire, a pediatric screening tool for ME/CFS in epidemiological studies in patients aged 5 to 17 years.

**Exposures:**

Seropositivity was determined by SARS-CoV-2 IgG antibodies in serum samples using enzyme-linked immunosorbent assays.

**Main Outcomes and Measures:**

Key symptoms of ME/CFS were evaluated separately or as clustered ME/CFS symptoms according to the DePaul Symptom Questionnaire, including fatigue.

**Results:**

Among 634 participants (294 male [46.4%] and 340 female [53.6%]; median age, 11.5 [IQR, 8-14] years), 198 (31.2%) reported clustered ME/CFS symptoms, including 40 of 100 SARS-CoV-2–seropositive (40.0%) and 158 of 534 SARS-CoV-2–seronegative (29.6%) children and adolescents. After adjustment for sex, age group, and preexisting disease, the risk ratio for reporting clustered ME/CFS symptoms decreased from 1.35 (95% CI, 1.03-1.78) to 1.18 (95% CI, 0.90-1.53) and for substantial fatigue from 2.45 (95% CI, 1.24-4.84) to 2.08 (95% CI, 1.05-4.13). Confinement to children and adolescents with unknown previous SARS-CoV-2 infection status (n = 610) yielded lower adjusted risks for all symptoms except joint pain ME/CFS–related symptoms. The adjusted risk ratio was 1.08 (95% CI, 0.80-1.46) for reporting clustered ME/CFS symptoms and 1.43 (95% CI, 0.63-3.23) for fatigue.

**Conclusions and Relevance:**

These findings suggest that the risk of ME/CFS in children and adolescents owing to SARS-CoV-2 infection may be very small. Recall bias may contribute to risk estimates of long COVID-19 symptoms in children. Extensive lockdowns must be considered as an alternative explanation for complex unspecific symptoms during the COVID-19 pandemic.

## Key Points

**Question:**

Is SARS-CoV-2 seropositivity associated with symptoms of myalgic encephalomyelitis and/or chronic fatigue syndrome (ME/CFS) in children and adolescents?

**Findings:**

This cross-sectional study of hospital-based SARS-CoV-2 seroprevalence surveys in Germany compared seropositive and seronegative children and adolescents and identified an excess of possible ME/CFS symptoms with serological evidence of preceding SARS-CoV-2 infection. This association almost disappeared with adjustment for confounders and restriction to children and adolescents unaware of preceding infection.

**Meaning:**

These findings suggest that the risk of ME/CFS after SARS-CoV-2 infection in children and adolescents may be small and that recall bias may contribute to risk estimates.

## Teaser

This cross-sectional study assesses the association between SARS-CoV-2 infection and symptoms related to myalgic encephalomyelitis and/or chronic fatigue syndrome (ME/CFS) in children and adolescents in Germany.

## Introduction

During the COVID-19 pandemic, an increase in nonspecific symptoms and mental health problems and a reduction in quality of life among children and adolescents have been reported.^1,2,3^ These outcomes could be attributable to infection or to containment measures such as social distancing, school closures, and restricted social activities owing to extensive lockdowns. In a German study,^1^ a substantial reduction in quality of life and mental health was already detected in the first months of the pandemic. With only approximately 2% of children and adolescents being infected at this time,^4^ it is reasonable to conclude that containment measures were at least partially responsible. During parts of the observation period, containment measures such as work-at-home mandates and reduction of social contacts were extended to full lockdown, including closures of shops and schools. The first nationwide lockdown with school closures was maintained from March 22 to April 15, 2020, with stepwise reopening of schools to May 6, 2020. The second nationwide lockdown, including school closures, was maintained from December 16, 2020, to March 3, 2021, when the government decided to gradually ease the lockdown measures depending on a stable incidence of less than 50 new infections per 100 000 inhabitants in a region.

Nonspecific somatic symptoms associated with long lockdown have been reported to overlap with key features of myalgic encephalomyelitis and/or chronic fatigue syndrome (ME/CFS), a complex disabling disease with symptoms of at least moderate severity and frequency persistent for at least 3 months, including fatigue, postexertional malaise, pain, sleep, and cognitive dysfunction as well as autonomous, neuroendocrine, and flulike symptoms.^5^ The etiology and pathomechanisms of ME/CFS are unclear, although infections by different pathogens are frequently reported triggers, and these symptoms are possibly mediated by postinfectious autoimmunity.^5,6,7,8^

It has been suggested that SARS-CoV-2 acts as a trigger for ME/CFS, and ME/CFS is considered to be a severe form of the long COVID-19 symptom complex.^9,10^ However, a clear causal link has not been established. In the absence of reliable biomarkers and causative factors for ME/CFS, epidemiological data may indicate associations with specific viral infections. Serological studies, including data on symptoms associated with ME/CFS, seem most appropriate to address this question. Serological data provide information on symptomatic and asymptomatic infections, thus representing the best available data source for a comparative risk assessment of ME/CFS–associated symptoms. Reported symptoms, however, may be prone to recall bias in children and adolescents who are aware of a preceding infection.

To assess the association between SARS-CoV-2 infection and ME/CFS–related symptoms among children and adolescents, we extended the cross-sectional seroprevalence surveys of SARS-CoV-2 IgG in Germany by items according to the Pediatric DePaul Symptom Questionnaire,^11^ a pediatric screening tool for ME/CFS in epidemiological studies. The DePaul Symptom Questionnaire was developed to achieve maximal screening sensitivity and not to establish a clinical diagnosis. Thus, a substantial proportion of cases reporting clustered ME/CFS symptoms are likely to represent false-positive results.

We hypothesized that there would be a difference between the proportion of children and adolescents reporting clustered ME/CFS symptoms according to the ME/CFS questionnaire and that for nonspecific individual symptoms possibly related to ME/CFS among seropositive and seronegative children and adolescents. To detect potential recall bias, we additionally considered awareness of a preceding SARS-CoV-2 infection.

## Methods

We performed a substudy of the SARS-CoV-2 KIDS study, which conducted hospital-based, multicenter cross-sectional surveys on the seroprevalence of anti–SARS-CoV-2 IgG antibodies in children in Germany from May 1 to October 31, 2021. Participants were recruited during their inpatient or outpatient stay in a pediatric hospital, regardless of the medical purpose of the stay. The ethics committee of each study center—including the Medical Faculty of Heidelberg University, Medical Faculty of Ludwig-Maximilians-University Munich, Technical University Dresden, North Rhine Medical Association, Albert-Ludwigs-University Freibug, Medical Faculty of the RWTH Aachen, Medizinische Hochschule Hannover, Julius-Maximilians University Wuerzburg, and Technical University Munich—approved the SARS-CoV-2 KIDS study and the amendments that were applied in our substudy. Parents or guardians of the children and adolescents provided written informed consent. This study followed the Strengthening the Reporting of Observational Studies in Epidemiology (STROBE) reporting guideline.

Serum samples of randomly selected pediatric patients collected for routine procedures were tested for IgG antibodies specific for the S1 domain of the SARS-CoV-2 spike protein using an enzyme-linked immunosorbent assay as described previously.^4^ Children with a corrected gestational age of less than 37 completed weeks, severe congenital or acquired immune deficiencies, immunosuppression due to chemotherapy or stem cell transplant, treatment due to life-threatening emergencies, and vaccination against SARS-CoV-2 were excluded from participation. Repeated participation was not possible.

A study-specific questionnaire provided clinical information. From May to October 2021, in 9 participating study centers, the parent study questionnaire on demographic and clinical information for children and adolescents aged 5 to 17 years was supplemented by screening questions for typical ME/CFS features such as frequent and persistent fatigue or extreme tiredness, pain, sleeping problems, cognitive difficulties, flulike symptoms, or disruptions in school activities. The questions were designed according to the DePaul Symptom Questionnaire.^11^ The English version of the questionnaire we used is provided in the eAppendix in the Supplement. Questions regarding the presence of symptoms referred to a period of 3 to 6 months in most questionnaires. Owing to a communication error, this period was limited to 3 months in a questionnaire that was used for a subset of 135 study participants. The definition of clustered ME/CFS symptoms implies reporting fatigue and/or school or cognitive difficulties and the experience of at least 4 additional symptoms.^11^ In addition to the algorithm proposed by Jason et al,^11^ we analyzed all symptoms individually. Symptoms were dichotomized if reported to be present for at least half of the time. According to Jason et al, fatigue was considered as fatigue or extreme tiredness that was present at least half of the time and was of at least moderate severity (defined hereinafter as *substantial fatigue*).

Patients who did not answer more than 2 questions about ME/CFS symptoms were excluded from the analysis. If 1 or 2 questions were missing, these were interpolated by the median value of the total cohort in this variable. Missing variables asking for the frequency of key symptoms were equally distributed across all questions, with a rate of 0.5% to 1.7% (*P* = .25) (eTable 1 in the Supplement). With 28 of 634 responses (4.4%) missing in the question about fatigue severity, the rate was slightly higher but was still less than 5% of all answers. Thus, a random distribution of missing values was ensured and no bias was caused by imputation for missing values.

### Statistical Analysis

We used χ^2^ tests and crude and multivariable logistic regression models. Multivariable regression models were adjusted for sex, age group, and preexisting disease—variables known to be significantly associated with the outcome and thus possible confounders. Preexisting conditions could be of any kind and were assumed if the question about preexisting conditions was answered with yes in the questionnaire. To evaluate potential subgroup effects, we calculated additional multivariable models for the 2 main outcomes of clustered ME/CFS symptoms and severe fatigue and included the additional variable federal state of the study center as a potential confounder.

We confined the analysis to children and adolescents with an unknown previous SARS-CoV-2 infection to identify potential recall bias. We defined a SARS-CoV-2 infection suggested by seropositivity as known in case of a recalled previous positive test result for SARS-CoV-2.

The severity of past COVID-19 illness could not be determined from the information in the questionnaire. Therefore, we only assessed whether the children had any respiratory diseases since the beginning of the pandemic in March 2020 (eg, fever and shortness of breath, pneumonia, fever for no obvious reasons, or hospitalization due to infection, pneumonia, or febrile illness). We investigated whether reporting of symptoms of any respiratory disease is associated with the 2 main outcomes of clustered ME/CFS symptoms and severe fatigue by calculating additional multivariable models stratified by SARS-CoV-2 serostatus. We defined children who never reported any respiratory infections as asymptomatic and others as symptomatic. Because the ME/CFS screening questions were implemented within the last months of the SARS-CoV-2 KIDS study only, we performed a post hoc power analysis for tests of 2 independent proportions.

The significance level was set a priori at 5% (*P* < .05). All statistical tests performed were 2-sided. We used SAS software, version 9.4 (SAS Institute Inc), for all statistical analyses.

## Results

After excluding participants with missing values (for sex, date of enrollment, date of birth, or >2 values in the long-COVID-19 part), data of 634 participants (294 male [46.4%] and 340 female [53.6%]; median age, 11.5 [IQR, 8-14] years) were included in the analysis (eFigure 1 and eTable 2 in the Supplement). Overall, 198 participants (31.2%) reported clustered ME/CFS symptoms, with a slightly higher proportion among children and adolescents reporting symptoms indicating ME/CFS throughout the past 3 to 6 months compared with the past 3 months (110 of 319 [34.5%] vs 88 of 315 [27.9%]; *P* = .07). Female sex (127 of 340 [37.3%] vs 71 of 294 [24.1%]; *P* < .001), older age (81 of 197 [41.1%] aged 14-17 years vs 117 of 437 [26.8%] aged 5-13 years; *P* < .001), and preexisting disease (110 of 300 [36.7%] vs 88 of 333 [26.4%]; *P* = .02) were associated with a higher probability of reporting clustered ME/CFS symptoms (eTable 2 in the Supplement).

One hundred of 634 participants (15.8%) were seropositive for SARS-CoV-2. Among the 100 seropositive participants, 40 (40.0%) reported clustered ME/CFS symptoms compared with 159 of the 534 seronegative participants (29.6%). After adjustment for sex, age group, and preexisting disease, the risk ratio (RR) for reporting clustered ME/CFS symptoms decreased from 1.35 (95% CI, 1.03-1.78) to 1.18 (95% CI, 0.90-1.53) (Table 1). Fatigue present at least half of the time and of at least moderate severity (substantial fatigue) was reported for 11 of the 100 seropositive participants (11.0%) and 24 of the 534 seronegative participants (4.5%). The unadjusted RR for substantial fatigue in seropositive children and adolescents was 2.45 (95% CI, 1.24-4.84); the adjusted RR, 2.08 (95% CI, 1.05-4.13). The inclusion of federal state of the study centers as an additional variable in the multivariable regression model changed the RR of clustered ME/CFS symptoms to 1.19 (95% CI, 0.92-1.56) and the RR of substantial fatigue to 2.12 (95% CI, 1.08-4.17).

**Table 1.** Association of SARS-CoV-2 Seropositivity in Children and Adolescents and Presence of Symptoms Associated With ME/CFS

Symptoms	Participant group, No. (%)		RR (95% CI)	
	Seropositive (n = 100)	Seronegative (n = 534)	Unadjusted	Adjusted^a^
Clustered ME/CFS symptoms^b^	40 (40.0)	158 (29.6)	1.35 (1.03-1.78)	1.18 (0.90-1.53)
Fatigue/extreme tiredness^c^				
Substantial fatigue	11 (11.0)	24 (4.5)	2.45 (1.24-4.84)	2.08 (1.05-4.13)
Symptoms suggesting school or cognitive difficulties^c^				
Missing activities because participant is too sick or too tired	6 (6.0)	32 (6.0)	1.00 (0.43-2.33)	0.84 (0.36-1.99)
Poor school attendance	5 (5.0)	32 (6.0)	0.83 (0.33-2.09)	0.70 (0.28-1.77)
Being unable or unwilling to go to school	7 (7.0)	21 (3.9)	1.78 (0.78-4.08)	1.52 (0.65-3.55)
School learning or memory problems	7 (7.0)	34 (6.4)	1.10 (0.50-2.41)	0.99 (0.45-2.19)
Additional symptoms^c^				
Headache	10 (10.0)	33 (6.2)	1.62 (0.82-3.18)	1.27 (0.64-2.50)
Sore throat	2 (2.0)	2 (0.4)	5.34 (0.76-37.47)	2.86 (0.41-19.88)
Joint pain	6 (6.0)	24 (4.5)	1.34 (0.56-3.18)	1.06 (0.44-2.52)
Muscle pain	4 (4.0)	15 (2.8)	1.42 (0.48-4.20)	1.11 (0.37-3.33)
Abdominal pain	7 (7.0)	39 (7.3)	0.95 (0.44-2.08)	0.68 (0.31-1.47)
Lymph node pain	1 (1.0)	2 (0.4)	2.67 (0.24-29.17)	2.75 (0.24-30.96)
Rash	4 (4.0)	9 (1.7)	2.37 (0.75-7.56)	2.20 (0.67-7.25)
Fever, chills, or shivers	2 (2.0)	6 (1.1)	1.78 (0.36-8.69)	1.91 (0.36-10.13)
Eye pain or light sensitivity	4 (4.0)	7 (1.3)	3.05 (0.91-10.23)	3.12 (0.91-10.74)
Problems sleeping	10 (10.0)	32 (6.0)	1.67 (0.85-3.28)	1.38 (0.69-2.74)
Impaired memory or concentration	6 (6.0)	32 (6.0)	1.00 (0.43-2.33)	0.80 (0.34-1.88)
Feeling worse, sick, or exhausted after exercise	8 (8.0)	43 (8.1)	0.99 (0.48-2.05)	0.79 (0.38-1.63)
Dizziness	5 (5.0)	8 (1.5)	3.34 (1.11-9.99)	2.72 (0.89-8.35)

Abbreviations: ME/CFS, myalgic encephalomyelitis and/or chronic fatigue syndrome; RR, risk ratio.

^a^ Adjusted for sex, age group, and preexisting disease.

^b^ Categorized as clustered ME/CFS symptoms implied reporting (1) substantial fatigue and/or symptoms of school or cognitive difficulties and (2) at least 4 additional symptoms.

^c^ All symptoms had to be present for at least the past 3 months and at least half of the time; if fatigue was present, it had to be at least moderate in severity.

Only 24 of 634 study participants (3.8%) reported having had past positive test results for SARS-CoV-2. Confinement to children and adolescents with an unknown previous SARS-CoV-2 infection status (n = 610) yielded lower adjusted risks for all ME/CFS–related symptoms (Table 2). In children and adolescents with an unknown previous SARS-CoV-2 infection, the adjusted RR for reporting clustered ME/CFS symptoms was 1.08 (95% CI, 0.80-1.46); the adjusted RR for substantial fatigue was 1.43 (95% CI, 0.63-3.23). When participants with remembered infection were excluded, the percentage decrease in adjusted risks was in the range of −8.5% to −70.8%, with little effect on reporting clustered ME/CFS symptoms (eTable 3 in the Supplement). The only exception was joint pain, with a small increase in the estimator (1.89%).

**Table 2.** Association of SARS-CoV-2 Seropositivity in Children and Adolescents and Presence of Symptoms Associated With ME/CFS Among Those With Unknown Previous SARS-CoV-2 Infection Status

Symptom	Infection status, No. (%)		RR (95% CI)	
	Seropositive (n = 82)	Seronegative (n = 528)	Unadjusted	Adjusted^a^
Clustered ME/CFS symptoms^b^	31 (37.8)	155 (29.3)	1.29 (0.95-1.75)	1.08 (0.80-1.46)
Fatigue/extreme tiredness^c^				
Substantial fatigue	7 (8.5)	24 (4.5)	1.88 (0.84-4.22)	1.43 (0.63-3.23)
Symptoms suggesting school or cognitive difficulties^c^				
Missing activities because participant is too sick or too tired	3 (3.7)	31 (5.9)	0.62 (0.20-1.99)	0.51 (0.16-1.65)
Poor school attendance	2 (2.4)	32 (6.1)	0.40 (0.10-1.65)	0.32 (0.08-1.33)
Unable or unwilling to go to school	3 (3.7)	21 (4.0)	0.92 (0.28-3.02)	0.71 (0.21-2.35)
School learning or memory problems	4 (4.9)	33 (6.3)	0.78 (0.28-2.15)	0.64 (0.23-1.78)
Additional symptoms^c^				
Frequent headaches	6 (7.3)	32 (6.1)	1.21 (0.52-2.80)	0.89 (0.39-2.07)
Sore throat	0	2 (0.4)	NA	NA
Joint pain	5 (6.1)	23 (4.3)	1.40 (0.55-3.58)	1.08 (0.42-2.79)
Muscle pain	3 (3.7)	15 (2.8)	1.29 (0.38-4.35)	0.97 (0.28-3.34)
Abdominal pain	5 (6.1)	39 (7.4)	0.83 (0.34-2.03)	0.55 (0.23-1.35)
Lymph node pain	0	2 (0.4)	NA	NA
Rash	0	9 (1.7)	NA	NA
Fever, chills, or shivers	1 (1.2)	5 (0.9)	1.29 (0.15-10.88)	1.50 (0.15-14.70)
Eye pain or light sensitivity	1 (1.2)	7 (1.3)	0.92 (0.11-7.38)	0.91 (0.11-7.56)
Problems sleeping	7 (8.5)	31 (5.9)	1.45 (0.66-3.19)	1.10 (0.50-2.44)
Impaired memory or concentration	4 (4.9)	30 (5.7)	0.86 (0.31-2.37)	0.64 (0.23-1.77)
Feeling worse, sick, or exhausted after exercise	4 (4.9)	42 (8.0)	0.61 (0.23-1.67)	0.47 (0.17-1.27)
Dizziness	2 (2.4)	8 (1.5)	1.61 (0.35-7.45)	1.16 (0.24-5.52)

Abbreviations: ME/CFS, myalgic encephalomyelitis and/or chronic fatigue syndrome; NA, not applicable; RR, risk ratio.

^a^ Adjusted for sex, age group, and preexisting disease.

^b^ Categorized as clustered ME/CFS symptoms implied reporting (1) substantial fatigue and/or symptoms of school or cognitive difficulties and (2) at least 4 additional symptoms.

^c^ All symptoms had to be present for at least the past 3 months and at least half of the time; if fatigue was present, it had to be at least moderate in severity.

The proportion of children and adolescents reporting respiratory symptoms was significantly lower in those who were unaware of their SARS-CoV-2 infection status (16 of 24 [66.7%]) compared with those reporting previous positive SARS-CoV-2 test results (137 of 610 [22.5%]; *P* < .001). Stratification of the analysis by SARS-CoV-2 serostatus showed that the adjusted RR for severe fatigue, depending on symptom status in the group of seropositive children, was 3.18 (95% CI, 1.11-9.11) compared with 2.44 (95% CI, 1.11-5.37) in the SARS-CoV-2–seronegative group (Table 3).

**Table 3.** Association of Symptom Status and Presence of Symptoms Associated With ME/CFS Stratified by SARS-CoV-2 Serostatus in Children and Adolescents

Symptom	Serostatus							
	SARS-CoV-2 seropositive (n = 100)				SARS-CoV-2 seronegative (n = 534)			
	No. (%) of participants		RR (95% CI)		No. (%) of participants		RR (95% CI)	
	Symptomatic^a^ (n = 31)	Asymptomatic (n = 69)	Unadjusted	Adjusted^b^	Symptomatic^a^ (n = 122)	Asymptomatic (n = 412)	Unadjusted	Adjusted^b^
Clustered ME/CFS symptoms^c^	15 (48.4)	25 (36.2)	1.34 (0.83-2.16)	1.31 (0.82-2.09)	52 (42.6)	106 (25.7)	1.66 (1.27-2.16)	1.78 (1.40-2.27)
Substantial fatigue^d^	6 (19.3)	5 (7.2)	2.67 (0.88-8.09)	3.18 (1.11-9.11)	9 (7.4)	15 (3.6)	2.02 (0.91-4.52)	2.44 (1.11-5.37)

Abbreviations: ME/CFS, myalgic encephalomyelitis and/or chronic fatigue syndrome; RR, risk ratio.

^a^ Defined as reporting any respiratory infection with symptoms of fever and shortness of breath, pneumonia, fever for no obvious reasons, or hospitalization due to infection, pneumonia, or febrile illness since the beginning of the COVID-19 pandemic in March 2020.

^b^ Adjusted for sex, age group, and preexisting disease.

^c^ Implies reporting (1) substantial fatigue and/or symptoms of school or cognitive difficulties and (2) at least 4 additional symptoms.

^d^ Implies fatigue and/or extreme tiredness that was present for at least the past 3 months, at least half of the time, and at least moderate in severity.

A power analysis of 100 exposed and 534 control participants and a failure rate of 0.3 among the controls yielded an effect estimate of 1.5 or higher to be detected with a power of at least 0.81. The power estimates by different relative risks are provided in eFigure 2 in the Supplement.

## Discussion

Comparison of SARS-CoV-2–seropositive and SARS-CoV-2–seronegative children and adolescents identifies an excess of key features of ME/CFS (clustered ME/CFS symptoms) and of substantial fatigue in those with serological evidence of preceding SARS-CoV-2 infections in the crude analysis. The risk estimates of these associations were reduced after adjustment for confounders and restriction to children and adolescents who were unaware of a preceding infection. An RR of less than 1.50 for reporting symptoms for ME/CFS triggered by a SARS-CoV-2 infection in children cannot be excluded based on the sample size of these data. The low RRs observed are in line with a preprinted report of routine health insurance data of a large matched German cohort revealing a statistically nonsignificant relative RR of 1.25 (95% CI, 0.24-6.65) for ME/CFS diagnosis (*International Statistical Classification of Diseases and Related Health Problems, Tenth Revision*, code G93.3) in children with vs children without previous COVID-19.^12^

As in the present study, the extent of unspecific symptoms in all children and adolescents during the COVID-19 pandemic reported by others^13^ might reflect an association with long lockdown. Our finding of an association between clustered ME/CFS symptoms and older age, female sex, and preexisting disease is consistent with findings of previous studies that have indicated a positive correlation between these parameters and persistent symptoms.^13^

Previous pediatric studies defining infected cases and controls by results of SARS-CoV-2 reverse transcriptase–polymerase chain reaction testing^14,15,16^ have reported a higher percentage of long COVID-19 symptoms in cases vs controls. One of these studies^15^ provided follow-up data that indicate a short duration of symptoms in most cases. However, these studies may be biased because of unrecognized infections and recall bias and therefore may overestimate the risk among children and adolescents.

Only a few pediatric studies^17,18,19^ have compared nonspecific symptoms in seronegative and seropositive children and adolescents. These investigators found slight differences in proportions of nonspecific symptoms between seropositive and seronegative patients, but none of these differences were statistically significant. In all pediatric studies comparing children with and without serological evidence of SARS-CoV-2 infection,^20^ the difference in prevalence in unspecific symptom burden between cases and controls was less than 4% and was statistically not significant.

A novel aspect of our analysis consists of using a validated screening tool for certain symptoms potentially indicating ME/CFS. By collecting information about the frequency of symptoms, it was possible to distinguish between occasional symptoms and those experienced more than half the time. Our findings revealed slightly increased unadjusted RRs for reporting clustered ME/CFS symptoms, substantial fatigue, and several additional symptoms in seropositive children and adolescents. However, adjustment for confounders and inclusion of knowledge about a preceding SARS-CoV-2 infection yielded decreased effect estimators.

### Limitations

This study has some limitations. The importance of serology as a marker of prior SARS-CoV-2 infection has been discussed among experts, and false-negative results are a factor in all studies based on serology. Because seroconversion does not provide information about the time of infection, the time span between infection and study participation cannot be determined. Therefore, the cross-sectional design does not consider the possible correlation between time of infection and occurrence of ME/CFS symptoms. Nevertheless, an advantage of this study design is that asymptomatic infections were also included in the analysis.

A further limitation pertains to external validity. The hospital-based study design may limit the representativeness of our sample in that children who did not require specialist outpatient or hospital care during the study period were not able to participate. Nevertheless, this study design allowed the inclusion of children and adolescents of all ages. Additionally, our findings may be limited by recall bias, which is a common feature among case-control studies.^21^ After restricting our analysis to patients who were unaware of their prior SARS-CoV-2 infection, the effect estimates for almost all outcomes were reduced markedly.

An alternative explanation for the reduction of effect estimates in children who lacked knowledge of a prior SARS-CoV-2 infection could be that recalled positive test results are associated with a high risk of symptomatic COVID-19. Unfortunately, the effects of COVID-19 severity cannot be evaluated using this data set owing to the lack of information about the pathogen responsible for the reported symptoms. The proportion of children reporting respiratory symptoms was significantly lower among participants who were unaware of their previous SARS-CoV-2 infection. However, the association of symptoms with the risk of clustered ME/CFS symptoms and severe fatigue was independent of serostatus. Thus, the reduction in the effect estimates of seropositivity as it pertains to the risk of clustered ME/CFS symptoms and severe fatigue could be attributed to recall bias. Although studies in adults have suggested that COVID-19 severity could be an independent risk factor for developing long COVID-19,^22^ our data suggest similar effect estimates of preceding symptomatic respiratory disease regarding the risk of clustered ME/CFS symptoms and severe fatigue irrespective of a confirmed SARS-CoV-2 infection. Indeed, many infectious diseases are known to cause persistent symptoms and infection-associated sequelae.^23,24^ The increased risk of fatigue among children reporting respiratory illness in the past may be attributable to SARS-CoV-2 infection as well as other infectious diseases. A recent study from Sweden reported an association between longer hospitalization and more severe persistent long COVID-19 symptoms among a small sample of children.^25^ However, rates of hospitalization for COVID-19–related therapy and severe courses with the need for intensive care treatment in children and adolescents are less than 1 in 1000,^26^ which may not be relevant with respect to our data.

## Conclusions

The findings of this cross-sectional study suggest that a symptom cluster that possibly indicates ME/CFS, as well as fatigue that is of at least moderate severity and is present approximately half of the time, are frequent features among children and adolescents with and without documented SARS-CoV-2 seroconversion. However, a small additional risk of ME/CFS symptoms associated with a preceding SARS-CoV-2 infection cannot be excluded owing to lack of power. Recall bias may account for overestimating the risk of long COVID-19 symptoms in any studies in which children were aware of their infection status. Without reliable biomarkers, it will remain difficult to differentiate long COVID-19, long lockdown, and many other diagnoses manifesting with unspecific symptoms among children and adolescents during the course of the COVID-19 pandemic.

## Notes

Supplement. **eTable 1.** Number of Participants With Missing Values per Question Asking for Key Symptoms Associated With Myalgic Encephalomyelitis and/or Chronic Fatigue Syndrome (ME/CFS) According to the DePaul Symptom Questionnaire **eTable 2.** Association of Demographic Characteristics With Reporting Clustered Myalgic Encephalomyelitis and/or Chronic Fatigue Syndrome (ME/CFS) Symptoms (n = 634) **eTable 3.** Comparison of Adjusted Risk Ratios for Symptoms Possibly Associated With Myalgic Encephalomyelitis and/or Chronic Fatigue Syndrome (ME/CFS) for All Children and Adolescents and Those With an Unknown Previous SARS-CoV-2 Infection **eFigure 1.** Flowchart of the Myalgic Encephalomyelitis and/or Chronic Fatigue Syndrome (ME/CFS) Screening Substudy of the SARS-CoV-2 KIDS Study **eFigure 2.** Estimated Power for the Outcome Reporting Clustered Myalgic Encephalomyelitis and/or Chronic Fatigue Syndrome (ME/CFS) Symptoms With Different Relative Risks Based on Pearson χ^2^ Test for Proportion Difference **eAppendix.** English Version of the 2-Page Study-Specific Questionnaire
