# Supplementary material for: Association of SARS-CoV-2 Seropositivity With Myalgic Encephalomyelitis and/or Chronic Fatigue Syndrome Among Children and Adolescents in Germany
Source: JAMA Netw Open. 2022 Sep 27;5(9):e2233454. doi: 10.1001/jamanetworkopen.2022.33454 (PMC9516317; doi:10.1001/jamanetworkopen.2022.33454)
Supplement: Supplement. — eTable 1. Number of Participants With Missing Values per Question Asking for Key Symptoms Associated With Myalgic Encephalomyelitis and/or Chronic Fatigue Syndrome (ME/CFS) According to the DePaul Symptom Questionnaire eTable 2. Association of Demographic Characteristics With Reporting Clustered Myalgic Encephalomyelitis and/or Chronic Fatigue Syndrome (ME/CFS) Symptoms (n = 634) eTable 3. Comparison of Adjusted Risk Ratios for Symptoms Possibly Associated With Myalgic Encephalomyelitis and/or Chronic Fatigue Syndrome (ME/CFS) for All Children and Adolescents and Those With an Unknown Previous SARS-CoV-2 Infection eFigure 1. Flowchart of the Myalgic Encephalomyelitis and/or Chronic Fatigue Syndrome (ME/CFS) Screening Substudy of the SARS-CoV-2 KIDS Study eFigure 2. Estimated Power for the Outcome Reporting Clustered Myalgic Encephalomyelitis and/or Chronic Fatigue Syndrome (ME/CFS) Symptoms With Different Relative Risks Based on Pearson χ2 Test for Proportion Difference eAppendix. English Version of the 2-Page Study-Specific Questionnaire [file jamanetwopen-e2233454-s001.pdf]

# Supplementary Online Content

Sorg AL, Becht S, Jank M, et al. Association of SARS-CoV-2 seropositivity with myalgic encephalomyelitis and/or chronic fatigue syndrome among children and adolescents in Germany. *JAMA Netw Open*. 2022;5(9):e2233454.
doi:10.1001/jamanetworkopen.2022.33454

**eTable 1.** Number of Participants With Missing Values per Question Asking for Key Symptoms Associated With Myalgic Encephalomyelitis and/or Chronic Fatigue Syndrome (ME/CFS) According to the DePaul Symptom Questionnaire

**eTable 2.** Association of Demographic Characteristics With Reporting Clustered Myalgic Encephalomyelitis and/or Chronic Fatigue Syndrome (ME/CFS) Symptoms (n = 634)

**eTable 3.** Comparison of Adjusted Risk Ratios for Symptoms Possibly Associated With Myalgic Encephalomyelitis and/or Chronic Fatigue Syndrome (ME/CFS) for All Children and Adolescents and Those With an Unknown Previous SARS-CoV-2 Infection

**eFigure 1.** Flowchart of the Myalgic Encephalomyelitis and/or Chronic Fatigue Syndrome (ME/CFS) Screening Substudy of the SARS-CoV-2 KIDS Study

**eFigure 2.** Estimated Power for the Outcome Reporting Clustered Myalgic Encephalomyelitis and/or Chronic Fatigue Syndrome (ME/CFS) Symptoms With Different Relative Risks Based on Pearson $\chi^2$ Test for Proportion Difference

**eAppendix.** English Version of the 2-Page Study-Specific Questionnaire

This supplementary material has been provided by the authors to give readers additional information about their work.

© 2022 Sorg AL et al. *JAMA Network Open*.

**eTable 1. Number of Participants With Missing Values per Question Asking for Key Symptoms Associated With Myalgic Encephalomyelitis and/or Chronic Fatigue Syndrome (ME/CFS) According to the DePaul Symptom Questionnaire**

| | **Number of participants with missing values** | ***P* value [a]** |
|---|---|---|
| | **No. (%)** | |
| Fatigue (frequency) | 8 (1.3) | 0.25 |
| Missing activities because he/ she is too sick or too tired | 3 (0.5) | |
| Poor school attendance | 10 (1.6) | |
| Being unable or unwilling to go to school | 7 (1.1) | |
| School learning or memory problems | 11 (1.7) | |
| Frequent headaches | 10 (1.6) | |
| Sore throat | 8 (1.3) | |
| Joint pain | 6 (0.9) | |
| Muscle pain | 9 (1.49 | |
| Abdominal pain | 10 (1.6) | |
| Lymph node pain | 0 (0) | |
| Rash | 5 (0.8) | |
| Fever, chills, or shivers | 5 (0.8) | |
| Eye pain or light sensitivity | 7 (1.19 | |
| Problems sleeping | 6 (0.9) | |
| Impaired memory, or concentration | 4 (0.6) | |
| Feeling worse, sick, or exhausted after exercise | 5 (0.8) | |
| Dizziness | 7 (1.1) | |

[a] According to $\chi^2$ testing

© 2022 Sorg AL et al. *JAMA Network Open*.

**eTable 2. Association of Demographic Characteristics With Reporting Clustered Myalgic Encephalomyelitis and/or Chronic Fatigue Syndrome (ME/CFS) Symptoms (n = 634)**

| | **Absolute numbers No. (%)** | **Clustered ME/CFS symptoms[a]** | | ***P* value[b]** |
|---|---|---|---|---|
| | | N | % [95% CI] | |
| Total | 634 (100) | 198 | 31.23% [27.61, 34.85] | |
| | | | | |
| Sex | | | | 0.0003 |
| Male | 294 (46.37) | 71 | 24.15% [19.24, 29.06] | |
| Female | 340 (53.63) | 127 | 37.35% [32.20, 42.51] | |
| | | | | |
| Age group, years | | | | <.0001 |
| 5 to 9 | 216 (34.07) | 45 | 20.83% [15.4, 26.26] | |
| 10 to 13 | 221 (34.86) | 72 | 32.58% [26.38, 38.77] | |
| 14 to 17 | 197 (31.07) | 81 | 41.12% [34.23, 48.01] | |
| | | | | |
| Migration background | | | | 0.8135 |
| No | 375 (59.15) | 118 | 31.47 [26.75, 36.18] | |
| Yes | 246 (38.8) | 77 | 31.3 [62.89, 74.51] | |
| Missing | 13 (2.05) | 3 | 1.0 [0.11, 46.04] | |
| | | | | |
| Pre-existing disease | | | | 0.0169 |
| No | 333 (52.52) | 88 | 26.43 [21.68, 31.18] | |
| Yes | 300 (47.32) | 110 | 36.67 [31.2, 42.13] | |
| Missing | 1 (0.16) | 0 | - | |
| | | | | |
| Federal state of the study center | | | | |
| Baden-Württember (Mannheim, Freiburg) | 242 (38.17) | 80 | 33.06 [27.11, 39.00] | 0.0274 |
| Bavaria (Munich, Würzburg) | 176 (27.76) | 50 | 28.41 [21.73, 35.09] | |
| Lower Saxony (Hannover) | 88 (13.88) | 38 | 43.18 [32.80, 53.56] | |
| Saxony (Dresden) | 61 (9.62) | 16 | 26.23 [15.16, 37.30] | |
| North Rhine-Westphalia (Krefeld, Aachen) | 67 (10.57) | 14 | 20.90 [11.13, 30.66] | |

[a] Categorized as clustered ME/CFS symptoms implied reporting 1) substantial fatigue and/or symptoms of school or cognitive difficulties and 2) at least 4 additional symptoms.
[b] According to $\chi^2$ testing

© 2022 Sorg AL et al. *JAMA Network Open*.

**eTable 3. Comparison of Adjusted Risk Ratios for Symptoms Associated With Myalgic Encephalomyelitis and/or Chronic Fatigue Syndrome (ME/CFS) for All Children and Adolescents and Those With an Unknown Previous SARS-CoV-2 Infection**

| | **All Participants** | **Participants unaware of previous SARS-CoV-2 infection.** | **Change of the adjusted RR in %** |
|---|---|---|---|
| | **Adjusted RR [95% CI][a]** | | |
| Clustered ME/CFS symptoms[b] | 1.18 [0.90, 1.53] | 1.08 [0.80; 1.46] | -8.47 |
| *Fatigue/extreme tiredness* [c] | | | |
| Substantial Fatigue | 2.08 [1.05; 4.13] | 1.43 [0.63, 3.23] | -31.25 |
| *Symptoms suggesting school or cognitive difficulties* [c] | | | |
| Missing activities because particpant is too sick or too tired | 0.84 [0.36, 1.99] | 0.51 [0.16, 1.65] | -39.29 |
| Poor school attendance | 0.70 [0.28, 1.77] | 0.32 [0.08, 1.33] | -54.29 |
| Unable or unwilling to go to school | 1.52 [0.65, 3.55] | 0.71 [0.21, 2.35] | -53.29 |
| School learning or memory problems | 0.99 [0.45, 2.19] | 0.64 [0.23, 1.78] | -35.35 |
| *Additional symptoms* [c]*:* | | | |
| Frequent headaches | 1.27 [0.64, 2.50] | 0.89 [0.39, 2.07] | -29.92 |
| Sore throat | 2.86 [0.41, 19.88] | NA | NA |
| Joint pain | 1.06 [0.44, 2.52] | 1.08 [0.42, 2.79] | 1.89 |
| Muscle pain | 1.11 [0.37, 3.33] | 0.97 [0.28, 3.34] | -12.61 |
| Abdominal pain | 0.68 [0.31, 1.47] | 0.55 [0.23, 1.35] | -19.12 |
| Lymph node pain | 2.75 [0.24, 30.96] | NA | NA |
| Rash | 2.20 [0.67, 7.25] | NA | NA |
| Fever, chills, or shivers | 1.91 [0.36, 10.13] | 1.50 [0.15, 14.70] | -21.47 |
| Eye pain or light sensitivity | 3.12 [0.91, 10.74] | 0.91 [0.11, 7.56] | -70.83 |
| Problems sleeping | 1.38 [0.69, 2.74] | 1.10 [0.50, 2.44] | -20.29 |
| Impaired memory, or concentration | 0.80 [0.34, 1.88] | 0.64 [0.23, 1.77] | -20.00 |
| Feeling worse, sick, or exhausted after exercise | 0.79 [0.38, 1.63] | 0.47 [0.17, 1.27] | -40.51 |
| Dizziness | 2.72 [0.89, 8.35] | 1.16 [0.24, 5.52] | -57.35 |

Abbreviations: ME/CFS, myalgic encephalomyelitis and/or chronic fatigue syndrome; NA, not applicable; RR, risk ratio.

[a] Adjusted for sex, age group, and pre-existing disease.

[b] Categorized as clustered ME/CFS symptoms implied reporting 1) substantial fatigue and/or symptoms of school or cognitive difficulties and 2) at least 4 additional symptoms.

[c] All symptoms had to be present for at least the past 3 months and at least half of the time; if fatigue was present, it had to be at least moderate in severity.

© 2022 Sorg AL et al. *JAMA Network Open*.

**eFigure 1. Flowchart of the Myalgic Encephalomyelitis and/or Chronic Fatigue Syndrome (ME/CFS) Screening Substudy of the SARS-CoV-2 KIDS Study**

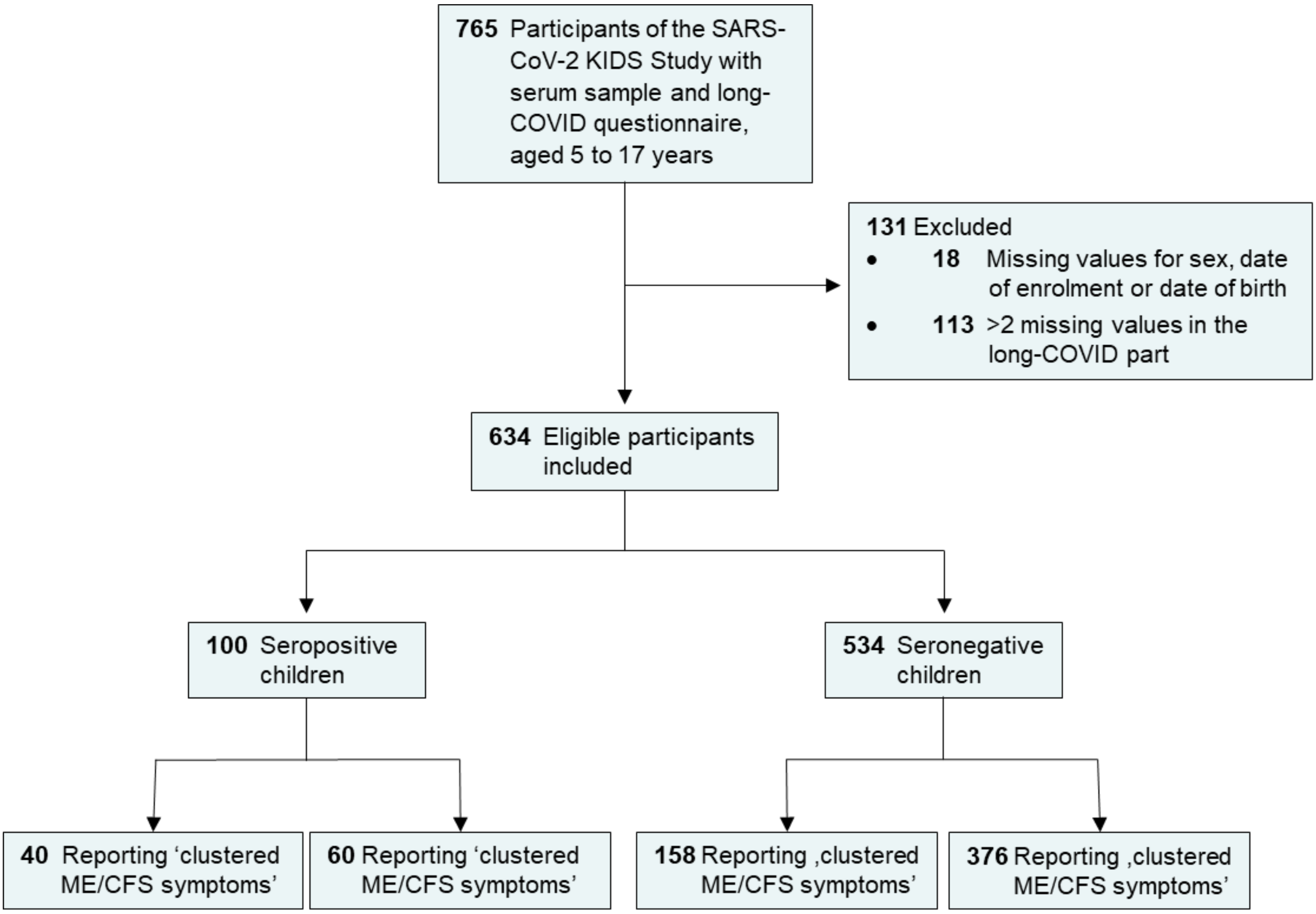


**Categorized as clustered ME/CFS symptoms' implied reporting substantial fatigue and/or symptoms of school or cognitive difficulties and at least 4 additional symptoms.**

© 2022 Sorg AL et al. *JAMA Network Open*.

**eFigure 2. Estimated Power for the Outcome Reporting Clustered Myalgic Encephalomyelitis and/or Chronic Fatigue Syndrome (ME/CFS) Symptoms With Different Relative Risks Based on Pearson $\chi^2$ Test for Proportion Difference**

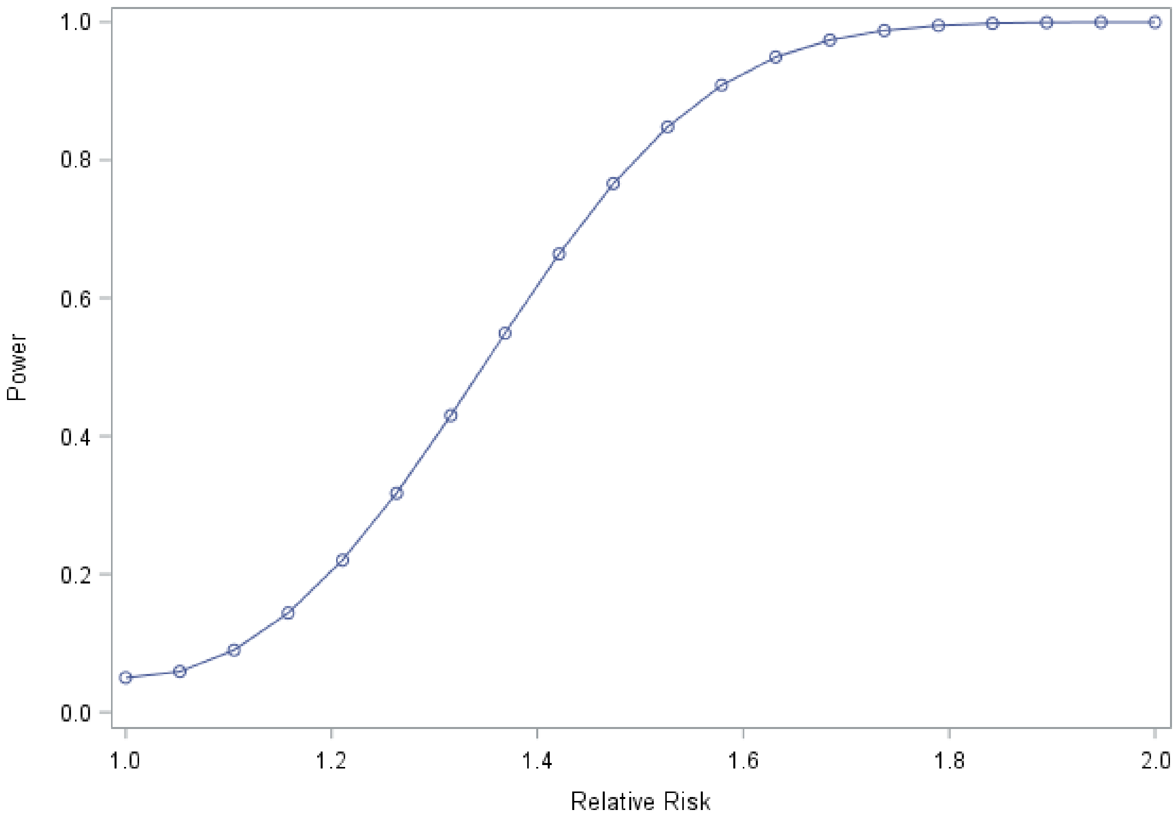


© 2022 Sorg AL et al. *JAMA Network Open*.

## eAppendix. English Version of the 2-Page Study-Specific Questionnaire

| (D) Questionnaire | Field for barcode sticker |
|---|---|

### 1. General Information

__ __ | __ __ | __ __ __ __
day month year

Please enter the date when the questionnaire was completed/ the blood draw was done.

Sex ☐ Female ☐ Male

Month of birth _ _
(example: month of birth January = 0 1)

Year of birth _ _ _ _
(example: year of birth 2014 = 2 0 1 4)

### 2. Country of origin of the parents

Mother's place of birth: ____________________

Father's place of birth: ____________________

Language spoken in the family:

☐ German ☐ Other: ______________________________

### 3. Reason for consulting a doctor in the hospital

What is the reason for the blood draw of your child?

☐ Planned treatment (surgery, MRI, Endoscopy etc.)
☐ Routine check-up (in the outpatient clinic)
☐ Referral for inpatient evaluation or parent/ patient education
☐ Emergency
☐ Another reason, which one? ____________________

Is your child currently being treated for a respiratory infection or pneumonia in the hospital?

☐ Yes ☐ No ☐ I do not know

### 4. Coronavirus (SARS-CoV-2)

Has your child ever been tested for SARS-CoV-2 (Coronavirus) in the past?

☐ Yes ☐ No ☐ I do not know

If Yes, what was the result of this test?
☐ Positive ☐ Negative ☐ I do not know

Have any close family members (shared household) tested **positive** for coronavirus in the past?

☐ No ☐ Yes, one close family member
☐ Yes, more than one close family member
☐ I do not know

### 5. Infectious diseases

How many respiratory infections⁺ with fever* and shortness of breath has your child gone through since March 2020 until today?

☐ None ☐ one ☐ two ☐ three ☐ four ☐ five
☐ six or more ☐ I do not know

*fever=body temperature >38°C ⁺A respiratory infection is a cold, bronchitis or flu

How often has pneumonia been diagnosed in your child since March 2020 until today? (please answer only if the diagnosis was made by a physician)

☐ never ☐ once ☐ twice ☐ more than twice
☐ I do not know

Did the pneumonia diagnoses require hospital treatment since March 2020?

☐ Yes ☐ No ☐ I do not know

→ If Yes, how often? ____ times

Did your child have fever for more than 48 hours since the beginning of March 2020 for no obvious reason?

☐ Yes ☐ No ☐ I do not know

If yes, were following symptoms present?

- Skin rash (exanthema) or skin/ mucosal inflammation ☐ Yes ☐ No ☐ I do not know
- Conjunctivitis ☐ Yes ☐ No ☐ I do not know
- Low blood pressure (hypotension) ☐ Yes ☐ No ☐ I do not know
- Circulation failure (shock) ☐ Yes ☐ No ☐ I do not know
- Features of myocardial dysfunction or heart inflammations/ abnormalities ☐ Yes ☐ No ☐ I do not know
- Blood clotting disorder (coagulopathy) ☐ Yes ☐ No ☐ I do not know
- Acute gastrointestinal problems (diarrhoea, vomiting, abdominal pain or suspected appendicitis) ☐ Yes ☐ No ☐ I do not know

Was your child hospitalized because of this febrile illness?

☐ Yes ☐ No ☐ I do not know

### 6. Underlying disease

Does your child have any pre-existing conditions?

☐ Yes ☐ No ☐ I do not know

Has your child ever had the following diseases?
(please tick as appropriate, multiple answers possible)

- Asthma ☐ Yes ☐ No ☐ I do not know
- Cystic fibrosis ☐ Yes ☐ No ☐ I do not know
- Bronchopulmonary dysplasia ☐ Yes ☐ No ☐ I do not know
- Another lung disease? Which one? ____________________
- Heart disease/ heart defect ☐ Yes ☐ No ☐ I do not know
- Haematological/ oncological disease ☐ Yes ☐ No ☐ I do not know
- Neurological/ neuromuscular disease ☐ Yes ☐ No ☐ I do not know
- Congenital or acquired immunodeficiency ☐ Yes ☐ No ☐ I do not know
- Autoimmune disease ☐ Yes ☐ No ☐ I do not know
- Metabolic disease ☐ Yes ☐ No ☐ I do not know
- Gastrointestinal disease ☐ Yes ☐ No ☐ I do not know
- Chronic renal disease ☐ Yes ☐ No ☐ I do not know
- Any other disease? ☐ Yes ☐ No ☐ I do not know
  Which one? ____________________

How often was your child hospitalized because of one of these underlying diseases in the past year?

☐ never ☐ once ☐ twice
☐ more than twice ☐ I do not remember

**Next on the back!** ↷

© 2022 Sorg A-L et al. *JAMA Network Open*.

***Only fill in the following questions, if your child is 5 years old or older***

## 7. Non-specific long-term effects of the Corona-Epidemic

| Please fill in the frequency and severity | **Frequency** – Throughout the past 3 (to 6) months, how often has your child had this symptom? | | | | | **Severity** – Throughout the past 3 (to 6) months, how much has this symptom bothered your child? | | | | |
|---|---|---|---|---|---|---|---|---|---|---|
| | None of the time | A little of the time | About half the time | Most of the time | All of the time | Symptom not present | Mild | Moderate | Severe | Very severe |
| Fatigue/ extreme tiredness | ❑ | ❑ | ❑ | ❑ | ❑ | ❑ | ❑ | ❑ | ❑ | ❑ |

| | Yes | No | Symptom not present |
|---|---|---|---|
| Has your child had a problem with fatigue or energy for **6 months or longer**? Please tick the right answer | ❑ | ❑ | ❑ |

| | Yes | No | I do not know |
|---|---|---|---|
| Did your child had problems with fatigue or low energy level **before the pandemic**? Please tick the right answer | ❑ | ❑ | ❑ |

| **Symptom**<br>Please tick the frequency for each symptom | **Frequency** – Throughout the past 3 (to 6) months, how often has your child had this symptom? | | | | |
|---|---|---|---|---|---|
| | None of the time | A little of the time | About half the time | Most of the time | All of the time |
| Missing activities because he/ she is too sick or too tired | ❑ | ❑ | ❑ | ❑ | ❑ |
| Poor school attendance (Even the occasional school offer is not taken up) | ❑ | ❑ | ❑ | ❑ | ❑ |
| Being unable or unwilling to go to school | ❑ | ❑ | ❑ | ❑ | ❑ |
| School learning or memory problems | ❑ | ❑ | ❑ | ❑ | ❑ |
| Frequent headaches | ❑ | ❑ | ❑ | ❑ | ❑ |
| Sore throat | ❑ | ❑ | ❑ | ❑ | ❑ |
| Joint pain | ❑ | ❑ | ❑ | ❑ | ❑ |
| Muscle pain | ❑ | ❑ | ❑ | ❑ | ❑ |
| Abdominal pain | ❑ | ❑ | ❑ | ❑ | ❑ |
| Lymph node pain (in neck or underarms) | ❑ | ❑ | ❑ | ❑ | ❑ |
| Rash | ❑ | ❑ | ❑ | ❑ | ❑ |
| Fever, chills, or shivers | ❑ | ❑ | ❑ | ❑ | ❑ |
| Eye pain or light sensitivity | ❑ | ❑ | ❑ | ❑ | ❑ |
| Problems sleeping | ❑ | ❑ | ❑ | ❑ | ❑ |
| Impaired memory, or concentration | ❑ | ❑ | ❑ | ❑ | ❑ |
| Feeling worse, sick, or exhausted after exercise | ❑ | ❑ | ❑ | ❑ | ❑ |
| Dizziness | ❑ | ❑ | ❑ | ❑ | ❑ |

# Thanks a lot for participating in this study!

Medical study direction:
Prof. Dr Horst Schroten
University Children's Hospital Mannheim
Theodor-Kutzer-Ufer 1-3 |
68167 Mannheim

Datacenter and statistics:
Prof. Dr. Rüdiger von Kries (retired)
LMU Munich - Institute for Social Pediatrics and Adolescent Medicine
Haydnstrasse 5 | 80336 Munich

Study coordination / Study lab
Prof. Dr. Christian Drosten
Charité University Medicine Berlin
Institute for Virology
Charitéplatz 1 | 10117 Berlin

© 2022 Sorg A-L et al. *JAMA Network Open*.
